# Longitudinal Comparison of Currently Used Risk Scores for Prognostication of Primary Sclerosing Cholangitis (PSC) in a Hungarian Bicenter PSC Cohort

**DOI:** 10.3390/diagnostics15172166

**Published:** 2025-08-26

**Authors:** Peter Laszlo Ven, David Tornai, Bence Toth, Zsuzsanna Vitalis, Istvan Tornai, Tamas Tornai, Gabriella Par, Maria Papp

**Affiliations:** 1 Division of Gastroenterology, First Department of Medicine, Medical School, University of Pécs, 7624 Pécs, Hungary; 2 Kálmán Laki Doctoral School of Biomedical and Clinical Sciences, Faculty of Medicine, University of Debrecen, 4032 Debrecen, Hungary; 3 Division of Gastroenterology, Department of Internal Medicine, Faculty of Medicine, University of Debrecen, 4032 Debrecen, Hungary; 4 European Reference Network on Hepatological Diseases, ERN RARE-LIVER, 4032 Debrecen, Hungary; 5 Institute of Pancreatic Diseases, Semmelweis University, 1089 Budapest, Hungary

**Keywords:** longitudinal assessment, prognostic scores, cholestatic disease, autoimmune liver disease (AILD), inflammatory bowel disease (IBD), epidemiology, composite outcome

## Abstract

**Background/Objectives:** Primary sclerosing cholangitis (PSC) is a chronic cholestatic liver disease with limited epidemiological data from Central–Eastern Europe. This study characterized a Hungarian PSC cohort, comparing patients with and without inflammatory bowel disease (IBD), and longitudinally evaluated the predictive efficacy of established prognostic scores (Mayo Risk Score, Amsterdam-Oxford Model [AOM], UK-PSC short/long). **Methods:** Data from 135 PSC patients (median diagnosis age 31 years, 57.7% male) were collected yearly at two Hungarian centers, with a median follow-up of 8.8 years. Outcomes included liver transplantation (LT) and liver-related death. Prognostic value of baseline clinical scores was assessed for 2-, 5-, 8-, and 10-year composite outcome. **Results:** PSC-IBD patients (54.1%) were younger with higher baseline Mayo and AOM scores, and had increased rates of colorectal carcinoma (8.22% vs. 0.00%) and liver transplantation (26.03% vs. 9.68%) within 10 years than PSC-only patients. There were no differences in liver-related mortality or composite outcomes between the groups. All prognostic scores showed good short-term predictive ability for poor outcomes (AUROC at 2 years: 0.858–0.958), which diminished over time (AUROC at 10 years: 0.708–0.756). The AOM demonstrated the most consistent performance. Persistent alkaline phosphatase (ALP) elevation (≥2.2×ULN) 2 years post-diagnosis, despite ursodeoxycholic acid therapy, strongly predicted 10-year adverse outcomes (HR: 3.927, *p* < 0.001), outperforming formal scoring systems (HR: 2.688–1.522). **Conclusions:** While PSC-IBD patients had more CRC and liver transplantation, overall transplantation-free survival was similar to PSC-only patients. Prognostic utility of current scores declines with longer follow-up; AOM was most stable. Sustained ALP elevation is a robust long-term prognostic indicator.

## 1. Introduction

Primary sclerosing cholangitis (PSC) is a rare, chronic cholestatic liver disease characterized by progressive inflammation and fibrotic obliteration of the intra- and/or extrahepatic bile ducts. Over time, this destructive cholangiopathy leads to biliary cirrhosis, portal hypertension, and ultimately liver failure in many patients [1,2]. PSC is also strongly associated with an increased risk of hepatobiliary and colorectal malignancies, particularly cholangiocarcinoma (CCA) and colorectal cancer (CRC), the latter primarily among those with concomitant inflammatory bowel disease (IBD) [3,4]. Despite its rarity, PSC remains a leading indication for liver transplantation in Europe and North America [5,6,7]. The epidemiology of PSC varies geographically. The highest reported incidence rates (1.0–1.5 per 100,000 person-years) are seen in Northern Europe and North America [4,8,9] while Southern and Central–Eastern European countries report lower incidence and prevalence figures [10,11,12,13]. However, these findings are primarily based on selected patient cohorts from Poland and Austria, rather than population-based epidemiological studies. The current literature is heavily weighted toward populations in Northwestern Europe and the United States, with a notable scarcity of epidemiological and clinical data from Central–Eastern Europe. Given the potential for regional variation in disease expression, comorbidities, and outcomes, additional studies in underrepresented populations are essential.

Risk stratification is also crucial in PSC to identify high-risk patients and guide surveillance, therapy, and transplantation decisions. However, there is currently no universally accepted method for estimating prognosis at the time of PSC diagnosis. The EASL Clinical Practice Guidelines (CPG) outline favorable and unfavorable phenotypic, laboratory, and imaging features that may assist non-invasive risk stratification, although their predictive value varies [8]. Trivedi et al. proposed a risk classification framework based on clinical symptoms, biochemical parameters, liver stiffness measurements, and biliary abnormalities on MRI/MRCP, enabling early identification of patients at significant risk for hepatic decompensation, liver transplantation, or liver-related death [14,15].

Alkaline phosphatase (ALP), due to its accessibility and reproducibility, has been extensively studied as a prognostic biomarker [16,17,18]. Although persistently elevated ALP levels are associated with poorer outcomes, the enzyme’s natural variability and inconsistent cut-off thresholds across studies limit its standalone utility. Consequently, current guidelines do not support ALP as a singular prognostic tool [8].

Historically, risk stratification in PSC has relied on general liver disease models such as the Child–Pugh score and the Model for End-Stage Liver Disease (MELD), both of which are limited by their focus on end-stage liver dysfunction and poor performance in early disease stages.

To meet this clinical need, several PSC-specific prognostic models have been developed in the last three decades (Wiesner-1989 [19], Farrant-1991 [20], Broome-1996 [21], revised Mayo Risk Score-2000 [22], Boberg-2002 [23], Ponsioen-2002 [24], Tischendorf-2007 [25]).

The Revised Mayo Risk Score (rMRS) was designed to predict short-term mortality using simple clinical and biochemical parameters. More recently, the Amsterdam-Oxford Model (AOM) [26], the UK-PSC risk scores (for short-term and long-term) [27], and the PSC Risk Estimate Tool (PREsTo) [28] have expanded the range of predictors and time horizons covered. Prognostic systems fundamentally aim to estimate transplant-free survival—except for PREsTo that predicts decompensation—but the clinical and laboratory parameters used show some differences (Table 1). Both the UK-PSC and AOM have proven to be reliable [29,30], and were also shown to be prognostic in recurrent cases [31]. However, only a limited number of comparative studies have been conducted.

This study provides epidemiological data from the Central–Eastern European region, while also evaluating and comparing the performance of major PSC-specific risk scores over time, and explores the prognostic utility of ALP in a Hungarian bicenter cohort.

## 2. Patients and Methods

### 2.1. Patient Population

The study was conducted retrospectively in two Hungarian tertiary gastroenterology centers of University of Debrecen and University of Pécs in accordance with the Declaration of Helsinki and approved by the Regional and Institutional Research Ethics Committees of University of Debrecen and University of Pécs (12759-6/2019/EÜIG, 9335-PTE2022—9 October 2022).

The diagnosis of PSC was established by MRCP in cases of elevated cholestatic enzymes, in accordance with the EASL recommendation [8]. In the absence of radiological abnormalities and suspected small bile duct disease, histological sampling was performed to establish the diagnosis. The diagnosis of autoimmune hepatitis (AIH) variant syndrome was made in patients meeting the diagnostic criteria for both PSC and AIH [32]. Diagnosis of coexisting IBD is based on clinical, endoscopic and histological findings as recommended by the ECCO-ESGAR guideline [33]. Laboratory results were always compared to the reference value of the center at the time. The clinical, radiological or endoscopic appearance of varices, ascites or hepatic encephalopathy was evaluated as decompensated cirrhosis.

### 2.2. Study Design

Between 1 January 2001, and 30 April 2019, a total of 135 patients were diagnosed with PSC at the two participating centers and included in the study. The follow-up period for the study concluded on 31 December 2023. The recording of patients’ basic demographic data and laboratory and imaging results started at the time of diagnosis and continued until liver transplantation, death, or the end of follow-up. The date of diagnosis was considered to be the date of MRCP or histological findings showing PSC-specific pathological abnormalities. The primary endpoint was time to liver-related death or liver transplantation. Demographic data included age, sex, height, and weight. Coexisting IBD and its subtype, AIH variant syndrome, and comorbidities were also determined. Biochemical, serological, imaging (ultrasound, CT, MRCP, ERCP, transient liver elastography) and histological results were collected. ALP cut-offs were defined based on the study of Goode et al. [27]. Regular outpatient visits were performed on an annual basis, with an increased frequency in case of progression and therapy adjustment. Laboratory tests were performed at least once a year. Data on IBD, presence, development or decompensation of cirrhosis (ascites, hepatic encephalopathy, variceal bleeding), and presence or development of various malignancies were collected during follow-up along with data on medical treatment, transplantation, and mortality. The rMRS, the MELD score, the UK-PSC score, AOM, and the PREsTo were determined at diagnosis and annually during follow-up. Considering the prognostic periods covered by risk stratification systems, in our study, we compared the systems based on the cohort results at follow-up intervals of 2, 5, 8, and 10 years.

### 2.3. Statistical Methods

Continuous patient characteristics were summarized as median and interquartile range (IQR). For categorical variables, frequencies were examined and presented as *n* (%). Variables were tested for normality using Shapiro–Wilk W test. Mann–Whitney U test was used to compare continuous variable levels between 2 groups. Kruskal–Wallis test or one-way ANOVA with a post hoc test for multiple comparisons was used to compare 3 groups as appropriate according to the distribution of the variables. The efficacy of different parameters to discriminate between outcomes was estimated by the ROC curve analysis. AUROC with corresponding 95% confidence intervals (CIs) and *p* values were calculated. The association between clinical variables and outcomes during follow-up was assessed with univariable Cox regression. Associations are provided as HR with 95% CIs. A 2-sided *p* value < 0.05 was considered statistically significant. For statistical analyses and graphical presentation, SPSS 29.0 (IBM, Armonk, NY, USA) and GraphPad Prism 10.4.1 (GraphPad Software, Boston, MA, USA) programs were used.

## 3. Results

### 3.1. Clinical Characteristics

The cohort included 135 patients (57.7% male), with a median age of 31 years at diagnosis (IQR: 20–47). The median follow-up duration was 105 months (IQR: 61–162). A total of 92 patients were diagnosed with large bile duct disease, 13 with small bile duct disease, and 30 with both large and small bile duct involvement. Coexisting IBD was present in 54.1% (43% ulcerative colitis, 10.4% Crohn’s disease, 0.7% unclassified), of which 80.8% were diagnosed prior to the PSC diagnosis. PSC-AIH overlap was present in 12.5%. At diagnosis, 17.7% were asymptomatic; others presented with abdominal pain (20.7%), pruritus (13.3%), weight loss (8.9%), and fatigue (8.1%). Ursodeoxycholic acid (UDCA) therapy was initiated in 85% and increased to 95.5% during follow-up. Patients’ clinical characteristics and admission laboratory results and scores are summarized in Table 2 and Table 3.

### 3.2. Follow-Up Outcomes

The median time to study endpoints (death, liver transplant, or end of the study period) was 105 months. Liver cirrhosis was present at baseline in 14.1% and developed in 22.9% during follow-up. Hepatic decompensation occurred in 32 patients (23.7%). During follow-up, 20 patients (14.8%) experienced bacterial infections requiring hospitalization. Osteoporosis was diagnosed in 12 patients (8.9%). A spectrum of malignancies was also observed, including cholangiocarcinoma in 6 patients (4.4%), ampullary (Vater’s papilla) carcinoma in 2, colorectal carcinoma in 6 (4.4%), and hepatocellular carcinoma (HCC) in 3 (2.2%). Additionally, isolated cases of various extrahepatic malignancies were identified, including endometrial adenocarcinoma, prostate cancer, adrenocortical carcinoma, papillary thyroid carcinoma, minimally invasive lung adenocarcinoma, ovarian carcinoma, and glioblastoma.

A total of 25 patients (18.5%) underwent liver transplantation, with 24 cases attributed to end-stage liver disease and 1 to hepatocellular carcinoma. Overall, 21 deaths (15.5%) were recorded during the study period. Of these, 15 (71.4%) were directly related to progression of hepatobiliary disease, including 3 associated with hepatobiliary malignancies. A total of 3 patients died due to colorectal cancer, while the remaining 3 deaths were attributed to unrelated causes. Additionally, 3 post-transplant deaths occurred, all due to sepsis.

At diagnosis, relatively little difference was observed between the PSC-only and PSC-IBD groups. Members of the PSC-IBD group were younger (26 yrs [IQR: 16–26] vs. 40 yrs [18,19,20,21,22,23,24,25,26,27,28,29,30,31,32,33,34,35,36,37,38,39,40]) and had significantly lower Mayo Risk Score (−1.82 [−2.54–−1.82] vs. −2.07 [−2.8–−2.07], *p* = 0.0187) and Amsterdam-Oxford Model values (1.13 [0.57–1.13] vs. 0.92 [0.13–0.92], *p* = 0.0151). However, there was no significant difference in composite outcome between the two groups (*p* = 0.3331). Outcome events in PSC-only and PSC-IBD groups are summarized in Table 4.

**Figure 1:**
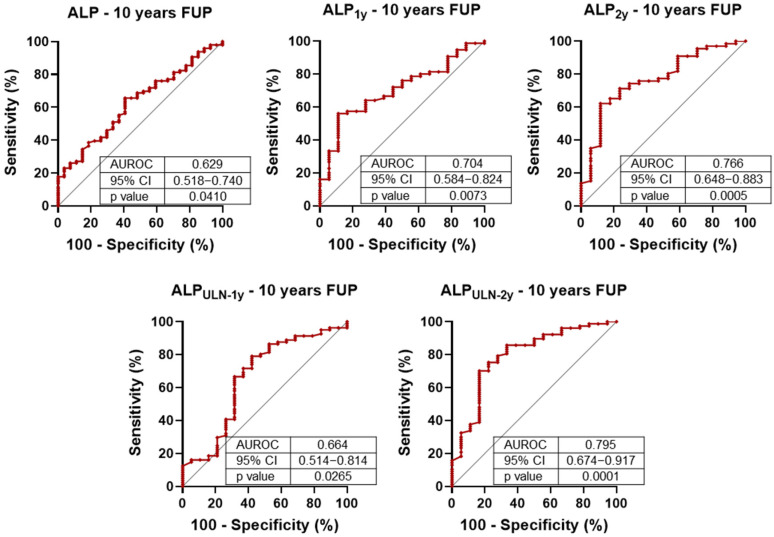
Receiver operating characteristic (ROC) analysis of alkaline phosphatase (ALP) levels for 10-year composite outcome (liver-related death and liver transplantation). ALP concentrations were assessed at baseline, 1 year (1y), and 2 years (2y). The discriminatory performance of ALP at 1 and 2 years was also evaluated relative to the upper limit of normal (ULN). AUROC: area under the ROC curve; CI: confidence interval.

We next evaluated the prognostic value of ALP levels measured at diagnosis, and at 1-year and 2-year follow-up visits, in relation to the 10-year composite endpoint. Despite the known intra-individual variability of ALP, ROC curve analysis demonstrated statistically significant associations at all time points (Figure 1). Notably, ALP measured at the 2-year mark—both as an absolute value and when normalized to the upper limit of normal (ULN)—consistently yielded the highest AUROC values, outperforming earlier measurements.

This trend was corroborated by univariate Cox regression analysis (Table 5): while ALP at diagnosis and at 1 year did not reach statistical significance, 2-year ALP levels were significantly associated with 10-year outcomes. Specifically, ALP levels exceeding 2.4 times the ULN at 1 year and 2.2 times the ULN at 2 years were associated with marked increases in hazard ratios, indicating improved predictive discrimination when using these cut-offs [27].

**Figure 2:**
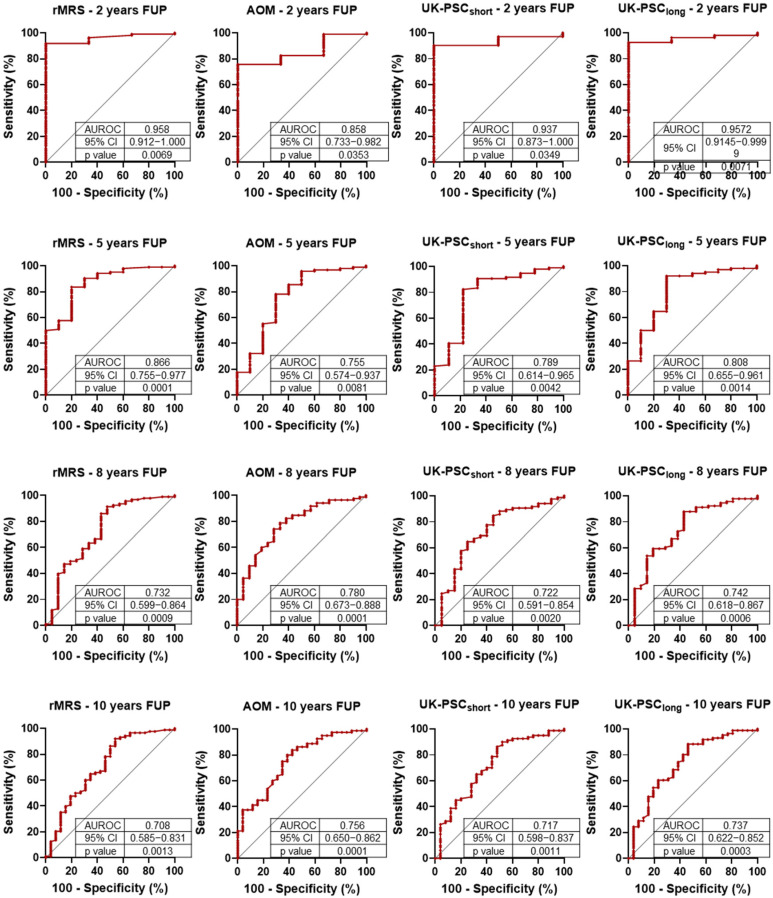
Receiver operating characteristic (ROC) analysis of primary sclerosing cholangitis (PSC)-related prognostic scores for 2-, 5-, 8- and 10-year composite outcomes (liver-related death and liver transplantation). rMRS: Revised Mayo Risk Score; AOM: Amsterdam-Oxford Model; FUP: follow-up; AUROC: area under the ROC curve; CI: confidence interval.

Next, we longitudinally analyzed the prognostic value of currently used risk assessment systems (rMRS, AOM, UK-short, and UK-long) for 2-, 5-, 8-, and 10-year composite outcome. The results of the ROC analysis are presented in Figure 2. All scores demonstrated good AUROC values (>0.7) in all examined years. The highest result was observed in every case in the 2nd year, followed by declining AUROC values over time.

The Cox proportional hazard regression (Table 6) provided similar results to the ROC analyses. All risk assessment scoring systems predicted the composite outcome for every investigated year. The greatest HR values were observed in the 2nd year, followed by declining trends during further follow-up. The UK-PSC-short score demonstrated the poorest predictive power while the AOM showed the most consistent results over time.

## 4. Discussion

Over the past several decades, numerous population-based studies have characterized the epidemiology and natural history of PSC in North America and Northwestern Europe [21,34,35,36,37,38,39,40,41,42,43]. In contrast, data from other global regions—including Central and Eastern Europe—remain limited. Our study addresses this regional data gap by providing the first population-based clinical and prognostic insights into PSC from a Hungarian cohort. Among Central European countries, most previously available epidemiologic insights originate from Austria and Poland, with findings largely consistent across studies regarding sex distribution, mean age at diagnosis, and the prevalence of IBD and cirrhosis [10,11,12,44]. However, compared to certain Polish cohorts, our study observed a lower prevalence of IBD and fewer patients undergoing liver transplantation [10,11,44]. These discrepancies may stem from differences in study design and selection criteria, as prior investigations were not explicitly epidemiological in nature. In our cohort, 54.1% of patients had concurrent IBD, a lower rate than the >60% typically reported in Western European cohorts [45]. Nonetheless, IBD preceded the diagnosis of PSC in over 80% of these patients, consistent with existing literature [6]. The higher transplantation rate in PSC-IBD patients may reflect earlier recognition and/or more proactive management rather than intrinsically worse liver disease, as cirrhosis development rates were similar between groups. While a few studies reported worse outcomes in patients with PSC-IBD [46] or specifically PSC-UC [47], most studies demonstrate comparable outcome rates regardless of IBD status [48,49,50]. Consistently, our study found similar composite outcome rates between groups, supporting the role of timely transplantation.

Cholangiocarcinoma (CCA) was identified in 4.4% of patients, which is at the lower end of the 8–13% range reported in large cohorts. However, this rate aligns with findings from several European studies reporting similarly low CCA incidences, such as those from Austria (3.3%), France (4.0%), Italy (2.2%), and Finland (4.5%) [12,36,51,52]. In contrast, studies from other Western European and Scandinavian countries tend to report higher CCA rates among patients with PSC. Notably, a large international study involving 37 centers across 17 countries found a CCA incidence of only 7.48%, further supporting the presence of regional variation [47]. This geographic discrepancy suggests that underlying genetic, environmental, lifestyle, or healthcare system-related factors may influence the detected incidence of CCA in PSC patients. Further studies are warranted to clarify the determinants of these regional differences, including the potential impact of varying screening practices, diagnostic approaches, and biological predispositions. There were no significant differences in CCA occurrence between PSC-only and PSC-IBD patients. However, colorectal cancer occurred exclusively in the PSC-IBD group, reinforcing its strong association with long-standing colonic inflammation in this population [53]. PSC-AIH overlap syndrome occurs in 7-14% of cases [8], which is in line with our data (12.5%). Only 2 (11%) of the PSC-AIH patients were confirmed to have small duct PSC, which is significantly lower than expected [8].

ALP, despite its high variability, is well-suited for prognostic purposes when interpreted in the appropriate context. In addition to the development of the UK-PSC scoring system, Goode and colleagues also formulated the independent predictive function of ALP in their study. Improved transplant-free survival results were found, when ALP decreased to <2.4 × ULN and <2.2 × ULN at 1 and 2 years following diagnosis, respectively [27]. Our study validates these findings, demonstrating that persistent ALP elevation despite UDCA treatment —considering these cut-off values—can serve as a valuable marker for transplantation and mortality risk assessment tools. Moreover, in our cohort, this single biomarker outperformed composite scores in predicting 10-year composite outcome. Notably, new therapies are being investigated for reducing ALP levels in PSC including fenofibrate [54], elafibranor (a dual peroxisome proliferator-activated receptor-α/δ agonist) [55], and cenicriviroc (a dual antagonist of C-C chemokine receptor types 2 and 5) [56]; however their long-term impact on survival still needs to be evaluated.

To date, few studies have compared PSC-specific prognostic scoring systems directly within a single cohort. A major strength of our study is the head-to-head, longitudinal evaluation of the most widely used models: the revised rMRS, the AOM, and the UK-PSC score. This allowed us to assess and contrast their short- and long-term predictive capacities over time. Validation studies of the newer prognostic scoring systems showed excellent predictive value for the UK-PSC score (AUROC: 0.81 and 0.80 for short- and long-term, respectively), while the Amsterdam-Oxford Model (AUROC: 0.67 (95% CI 0.64–0.70) also demonstrated reliable performance [27,28,57,58]. In a study investigating soluble macrophage markers, the rMRS and the AOM performed similarly for predicting 8-year transplant-free survival [59]. In our study, the rMRS had the highest early AUROC but declined more sharply over time. The AOM model showed the most stable performance and may be particularly well-suited for long-term stratification. In our cohort, the UK-PSC short-term risk score demonstrated lower predictive performance compared to other prognostic models and to its originally published validation metrics. A decline in the accuracy of all scoring systems over extended follow-up was also observed. This temporal decrease in prognostic discrimination is likely multifactorial. In early disease stages—characterized by biochemical fluctuations and heterogeneous progression—it remains challenging to reliably estimate disease trajectory, even with sophisticated composite scoring systems. In contrast, once advanced liver disease manifests, clinical endpoints become more predictable and more easily captured by risk algorithms. These findings underscore the limitations of current models in forecasting long-term outcomes from early disease data and highlight the need for dynamic or longitudinally adaptive scoring approaches. Our results confirm the conclusion already suggested by the literature: despite their differences, all three scoring systems (RMS, UK-PSC, AOM) can be reliably used for risk assessment. In the ROC analysis, we did not observe significant differences between them; however, the similarity of the results may be influenced by the sample size of our cohort, which limits the granularity of the assessment.

In summary, our study provides data on the clinical profile and prognosis of PSC in Hungary addressing a key regional data gap in Central–Eastern Europe, and offers the first direct, longitudinal comparison of major PSC-specific prognostic models within the same cohort. It also confirms the utility of ALP, particularly persistent elevation above 2.2 × ULN, as a robust predictor of long-term outcomes. Despite its strengths, our study has some limitations including the retrospective design and the relatively small sample size. However, the population-based nature of the data might mitigate these limitations and enhance its real-world relevance.

## 5. Conclusions

The 10-year liver-related mortality or composite adverse outcomes of PSC were found to be independent of coexisting IBD, despite the higher number of transplanted patients in the PSC-IBD group in our large Hungarian cohort. However, colorectal carcinoma occurred exclusively in the IBD group. Our findings demonstrate that the prognostic accuracy of established PSC-specific scoring systems, including the rMRS, AOM, and UK-PSC models, diminishes over extended follow-up periods. Although the overall performance of these scores was comparable, the UK-PSC short model exhibited the lowest prognostic ability, whereas the AOM provided the most consistent predictive performance over time. Importantly, a persistent elevation in serum ALP levels two years after diagnosis, despite UDCA therapy, emerged as a more robust predictor of poor 10-year composite outcomes (liver transplantation or liver-related death) than any of the evaluated formal prognostic scores. These results underscore the importance of monitoring dynamic markers like ALP for long-term risk stratification in PSC patients and highlight the need for further improvement in current PSC-specific prognostic tools.

## Figures and Tables

**Table 1:** PSC-specific prognostic scoring systems contain similar parameters.

	Revised Mayo Risk Score	UK-PSC Short	UK-PSC Long	AOM	PREsTo
Age (yrs)	✓	✓	✓	✓	✓
PSC duration (yrs)					✓
Sodium (mmol/L)					✓
Bilirubin (mg/dL)	✓	✓	✓	✓	✓
Albumin (g/dL)	✓	✓	✓	✓	✓
AST (U/L)	✓			✓	✓
ALP (U/L)			✓	✓	✓
Hb (g/dL)		✓			✓
Platelets (×10^9^/L)		✓	✓	✓	✓
Variceal bleed	✓		✓		
Cholangiogram		presence of extrahepatic disease	presence of extrahepatic disease	small or large	
Outcome	Death	Death/LT	Death/LT	PSC-related death/LT	Hepatic decompensation
Range of the prognostication (years)	1–4	1–2	5; 8	1–15	1–5

✓ indicates that a variable is included in the score. AST: aspartate aminotransferase; ALP: alkaline phosphatase; Hb: hemoglobin.

**Table 2:** Baseline characteristics of the entire cohort.

Baseline Characteristics	PSC Patients (*N* = 135)
Male gender, *n* (%)	78 (57.7)
Age at diagnosis, median (IQR)	31 (20–47)
PSC-AIH, *n* (%)	17 (12.5)
Small duct PSC, *n* (%)	13 (9.6)
Coexisting IBD, *n* (%)	73 (54)
Ulcerative colitis, *n* (%)	58 (79.4)
Crohn’s disease, *n* (%)	14 (19.1)
ALP, median (IQR)	383 (199–647)

PSC-AIH: primary sclerosing cholangitis—autoimmune hepatitis variant syndrome; IBD: inflammatory bowel disease; ALP: alkaline phosphatase.

**Table 3:** Laboratory parameters and prognostic scores in PSC-only and PSC-IBD patients.

	PSC-Only (*n* = 62)	PSC-IBD (*n* = 73)	*p* Value
ALP	367.00 (106–367)	404 (149–404)	0.2697
Albumin	45 (30.8–45)	45 (38–45)	0.8652
INR	0.99 (0.88–0.99)	1.05 (0.94–1.05)	0.0549
Hemoglobin	138 (112.8–138)	134 (107–134)	0.1809
Cholinesterase	7.65 (3.37–7.65)	7.2 (5.08-7.2)	0.3093
rMRS	−1.82 (−2.54–−1.82)	−2.07 (−2.8–−2.07)	**0.0187**
AOM	1.13 (0.57–1.13)	0.92 (0.13–0.92)	**0.0151**
UK-PSC short	−8.05 (−9.05–−8.05)	−7.81 (−8.89–−7.81)	0.1633
UK-PSC long	−1.61 (-2.52–−1.61)	−1.84 (−2.7–−1.84)	0.1318
PREsTo 1. year	0.51 (0.36–0.51)	0.46 (0.36–0.46)	0.2556
PREsTo 2. year	1.00 (0.7–1)	0.90 (0.71–0.9)	0.2556
PREsTo 3. year	1.47 (1.03–1.47)	1.32 (1.04–1.32)	0.2556
PREsTo 4. year	2.37 (1.67–2.37)	2.14 (1.68–2.14)	0.2556
PREsTo 5. year	3.68 (2.59–3.68)	3.32 (2.68–3.32)	0.2545

Data are presented as median (IQR). Bold numbers indicate statistically significant *p* values. ALP: alkaline phosphatase; INR: international normalized ratio; rMRS: Revised Mayo Risk Score; AOM: Amsterdam-Oxford Model; PSC Risk Estimate Tool: PREsTo.

**Table 4:** Primary outcomes in PSC-only and PSC-IBD groups.

	PSC-Only (*n* = 62)	PSC-IBD (*n* = 73)	*p* Value
Cirrhosis development	22 (35.4%)	28 (38.3%)	0.7305
Cholangiocarcinoma development	3 (4.84%)	3 (4.11%)	0.8377
Hepatocellular carcinoma development	1 (1.61%)	2 (2.74%)	0.6580
Colorectal carcinoma development	0 (0.00%)	6 (8.22%)	**0.0308**
Other extrahepatic malignancy development	6 (9.68%)	8 (10.96%)	0.8077
Liver transplantation	6 (9.68%)	19 (26.03%)	**0.0148**
Death	11 (17.74%)	7 (9.59%) ^#^	0.2068
Liver-related death	8 (12.90%)	7 (9.59%)	0.5910
Composite endpoint *	14 (22.58%)	23 (31.51%)	0.3331

Data are presented as *n* (%). Bold numbers indicate statistically significant *p* values. ^#^ Additional 3 after liver transplantation. * Liver transplantation and liver-related death.

**Table 5:** Univariate Cox proportional hazard regression analysis of 10-year composite endpoint.

	HR	95% CI	*p* Value
(Ln) ALP at baseline	1.433	0.877–2.341	0.1514
(Ln) ALP at 1 year	1.682	0.953–2.969	0.0728
(Ln) ALP at 2 years	2.964	1.572–5.59	**0.0008**
(Ln) ALP × ULN at 1 year	1.943	0.948–3.985	0.0698
(Ln) ALP × ULN at 2 years	3.927	2.091–7.377	**<0.0001**
ALP > 2.4 × ULN at 1 year	3.325	1.308–8.456	**0.0116**
ALP > 2.2 × ULN at 2 years	7.972	2.306–27.56	**0.0010**

Bold numbers indicate statistically significant *p* values. ALP: alkaline phosphatase; ULN: upper limit of normal; HR: hazard ratio; CI: confidence interval; × ULN: times the ULN.

**Table 6:** Univariate Cox proportional hazard regression analysis of liver transplantation or death.

Name	FUP Year	HR	CI	*p* Value
rMRS	2	3.318	1.562–7.048	**0.002**
	5	2.523	1.644–3.871	**<0.001**
	8	2.269	1.544–3.333	**<0.001**
	10	2.195	1.524–3.161	**<0.001**
AOM	2	3.232	1.28–8.159	**0.013**
	5	2.696	1.576–4.612	**<0.001**
	8	2.723	1.747–4.243	**<0.001**
	10	2.688	1.782–4.055	**<0.001**
UK-PSC short	2	1.989	1.052–3.761	**0.034**
	5	1.638	1.195–2.245	**0.002**
	8	1.507	1.162–1.954	**0.002**
	10	1.522	1.204–1.924	**<0.001**
UK-PSC long	2	3.301	1.531–7.118	**0.002**
	5	2.274	1.493–3.463	**<0.001**
	8	2.056	1.455–2.907	**<0.001**
	10	2.049	1.492–2.813	**<0.001**

Bold numbers indicate statistically significant *p* values. HR: hazard ratio; CI: confidence interval; rMRS: Revised Mayo Risk Score; AOM: Amsterdam-Oxford Model

## Data Availability

The data presented in this study are available on request from the corresponding author. The data is not publicly available due to privacy restrictions.

## Abbreviations

The following abbreviations are used in this manuscript:

AIH	Autoimmune hepatitis
ALP	Alkaline phosphatase
AOM	Amsterdam-Oxford Model
AST	Aspartate aminotransferase
AUROC	Area under the receiver operating characteristic curve
CCA	Cholangiocarcinoma
CI	Confidence interval
CRC	Colorectal cancer
CT	Computed tomography
EASL	European Association for the Study of the Liver
CPG	Clinical Practice Guideline
ECCO	European Crohn’s and Colitis Organisation
ERCP	Endoscopic retrograde cholangiopancreatography
ESGAR	European Society of Gastrointestinal and Abdominal Radiology
FUP	Follow-up
Hb	Hemoglobin
HCC	Hepatocellular carcinoma
HR	Hazard ratio
INR	International normalized ratio
IQR	Interquartile range
IBD	Inflammatory bowel disease
LT	Liver transplantation
MELD	Model for End-Stage Liver Disease
MRI	Magnetic resonance imaging
MRCP	Magnetic resonance cholangiopancreatography
PREsTo	PSC Risk Estimate Tool
PSC	Primary sclerosing cholangitis
ROC	Receiver operating characteristic
rMRS	Revised Mayo Risk Score
UDCA	Ursodeoxycholic acid
ULN	Upper limit of normal
